# How can waist circumference predict the body composition?

**DOI:** 10.1186/1758-5996-6-11

**Published:** 2014-01-29

**Authors:** Yumi Matsushita, Toru Nakagawa, Michihiro Shinohara, Shuichiro Yamamoto, Yoshihiko Takahashi, Tetsuya Mizoue, Tetsuji Yokoyama, Mitsuhiko Noda

**Affiliations:** 1 Department of Clinical Research, National Center for Global Health and Medicine, 1-21-1 Toyama, Shinjuku-ku, Tokyo 162-8655, Japan; 2 Hitachi, Ltd. Hitachi Health Care Center, 4-3-16, Ose-cho, Hitachi-shi, Ibaraki-ken 317-0076, Japan; 3 Division of Diabetes and Metabolism, Iwate Medical University, 19-1 Uchimaru, Morioka, Iwate 020-8505, Japan; 4 Department of Epidemiology and Prevention, National Center for Global Health and Medicine, 1-21-1 Toyama, Shinjuku-ku, Tokyo 162-8655, Japan; 5 Department of Health Promotion, National Institute of Public Health, 2-3-6 Minami, Wako, Saitama 351-0197, Japan; 6 Department of Diabetes Research, National Center for Global Health and Medicine, 1-21-1 Toyama, Shinjuku-ku, Tokyo 162-8655, Japan

## Abstract

**Background:**

Waist circumference (WC) is used as a risk assessment for metabolic syndrome, diabetes, and cardiovascular disease (CVD). WC consists of visceral fat area (VFA), subcutaneous fat area (SFA), muscle, intramuscular fat, viscera, and bone. Each component of the WC may differ between the sexes and generations, even if they have the same WC. However, this has not been measured in an epidemiological study.

**Methods:**

Between 2004 to 2009, employees and their spouses working at a Japanese company underwent a health examination after more than 12 hours of fasting. We analyzed the data of 11,570 subjects (9,874 men and 1,696 women), aged from 20 to 76 years, who underwent a computed tomography (CT) examination. VFA, SFA, WC, muscle, intramuscular fat, viscera, and bone were measured using a CT scanner. We conducted stratified analyses by generational age, and calculated the Pearson’s correlation coefficients between the VFA and WC, BMI, and VFA plus SFA. To establish the equations for converting the WC to the corresponding VFA and VFA plus SFA, linear regression analyses were used to obtain the regression coefficients and intercepts.

**Results:**

As the generations increased in age, the VFA tended to increase. However, the differences in the WC values of each generation did not coincide with the VFA values in men (r = -0.275 and 0.979 for men and women, n = 5 generations), but did correlate with the difference in the sum of the VFA plus SFA for both sexes (r = 0.915 and 0.996 for men and women, n = 5 generations). Older generations had lower WC values when they had the same VFA values as the younger generations.

**Conclusions:**

The WC value corresponding to a certain VFA value differed significantly by generational age. Thus, revised optimal cutoff values for the WC may be needed for each generation.

## Background

Metabolic syndrome (MS) is characterized by central obesity, impaired glucose tolerance, high blood pressure, and abnormal lipid metabolism [1]. Individuals with MS have a higher risk of cardiovascular disease, and a subsequent increase in disease mortality and morbidity [2-4]. In 2008, the age-standardized death rates of cardiovascular disease per 100,000 in Japanese, Chinese, Indonesian, and American men were 121.3, 311.2, 186.8, and 366.1, respectively. The rates in Japanese, Chinese, Indonesian, and American women were 76.0, 263.0, 128.4, and 268.7, respectively [5]. The prevalence of MS defined by the modified National Cholesterol Education Program’s Adult Treatment Panel III (NCEP-ATP) guidelines [6] in Japan and China was 20.6% [7] and 27.6% [8], respectively.

In the diagnosis of MS, the waist circumference (WC) is almost always used as a criteria, and this measurement is typically used as a surrogate measure of the visceral fat area (VFA) [1,6,9,10]. A previous study demonstrated the prevalence of MS under the NCEP-ATP III definitions (the obesity index used BMI at more than 25, instead of WC) in Asian ethnic groups (Japan, Korea, and Mongolia) using the same research design and protocols for all subjects [11]. There were no significant differences in the prevalence of MS among the three Asian ethnic groups, in spite of great differences in BMI and in each metabolic parameter.

Several studies have shown a strong correlation between the VFA and anthropometric values, such as the BMI and WC [12,13], thus, these easily measurable anthropometric values have been used as surrogate indices of the VFA, regardless of age. Body composition can differ greatly with age [14,15] and it is unknown whether the relationships between the VFA and BMI or WC are the same throughout the different age groups. Previous studies have shown that visceral adipose tissue positively correlated with age in both men and women [13,16], but there were differences in the mean BMI [14] and the mean WC [14,15] among the different age groups. Differences in body components according to age has not yet been clarified in a large epidemiological study.

In this study, we investigated correlations of the VFA, measured by computed tomography (CT), with WC and BMI, and compared the mean VFA and subcutaneous fat area (SFA) according to gender and age groups. Furthermore, we analyzed the CT image divided into VFA, SFA, muscle, visceral and bones to examine how WC can predict body composition.

## Methods

### Participants and procedures

Employees and their spouses working at the Hitachi company, Ibaraki Prefecture, between 2004 to 2009, underwent a health examination after more than 12 hours of fasting. Of these participants, we analyzed the data for 11,570 subjects (9,874 men and 1,696 women), aged between 20 to 76 years, who underwent a CT examination. Body height and weight were measured using an automated scale (BF-220; TANITA), and the BMI was defined as the weight (kg) divided by the square of the height (m^2^). The VFA, SFA and WC were measured using a CT scanner and protocols described elsewhere [17]. Briefly, single slice imaging was performed at the umbilicus level in a supine position, using a Redix turbo CT machine (Hitachi Medico). The imaging conditions were 120 kV, 50 mA, and a slice thickness of 5 mm. The VFA, SFA and the WC were calculated using fatPointer software (Hitachi Medico). This system also divided and analyzed CT-scanned images into 5 areas (VFA, SFA, muscle, intramuscular fat, and viscera) and others (bone and air). The division process was performed in the following way: first, the body area was extracted from the CT-scanned abdominal image. Then, the specific body area without fat (epidermis, bone, muscle and visceral organs; external fat area) was extracted from the initial extracted body area. Next, the external fat area was segregated into the viscera and subcutaneous fat, based on the location area of the initial extracted body area. And then, areas of muscle, intramuscular fat, and visceral organs were segregated from the located information of the contained visceral and the external fat area. Finally, each separated area was analyzed and displayed in different colors. HbA1c levels were measured using a HPLC method with an ADAMS HA8160 device (Arkrey). Blood glucose levels were measured using the glucose electrode technique with an ADAMS glucose GA1170 device (Arkrey). Blood pressure was measured using an oscillometric method with a Kentaro ADVANCEBP-203RV III A/B device (Colin) while the patient was in a sitting position and after the patient had rested for 3 min. Informed consent was obtained from each examinee regarding the use of his or her data for research purposes. The present study protocol was approved by the ethics review committee of the National Center for Global Health and Medicine.

### Statistical analyses

All analyses were performed according to gender. We conducted stratified analyses by generational age (under 39, 40–49, 50–59, 60–69, and over 70 years). To compare the characteristics of the subjects in the five age groups, ANOVA or regression analysis was used for the continuous variables, such as VFA, SFA, WC, BMI, and laboratory values. We calculated the Pearson’s correlation coefficients between the VFA and WC, BMI, and VFA plus SFA. We also calculated the Pearson’s correlation coefficients between the VFA plus SFA and the anthropometric indices (WC and BMI). To establish the equations for converting the WC to the corresponding VFA and VFA plus SFA, linear regression analyses were used to obtain the regression coefficients and intercepts. All analyses were performed using SPSS software for Windows, Version 15.0 (SPSS Inc., IL, USA).

## Results

The characteristics of the subjects are shown in Table 1. The mean age of the study subjects was 52.8 ± 10.0 years for men and 57.2 ± 9.5 years for women. The mean BMI was 24.1 ± 3.0 kg/m^2^ in men and 23.1 ± 3.5 kg/m^2^ in women. The mean VFA was 123.2 ± 53.5 cm^2^ in men and 84.4 ± 47.1 cm^2^ in women. The mean WC was 86.6 ± 11.4 cm in men and 83.8 ± 9.5 cm in women. The mean HbA1c was 5.8 ± 0.7% in men and 5.8 ± 0.8% in women. The mean fasting plasma glucose was 106.5 ± 20.9 mg/dL in men and 100.9 ± 20.1 mg/dL in women. The mean systolic blood pressure was 121.7 ± 12.3 mmHg in men and 119.9 ± 14.3 mmHg in women. The mean diastolic blood pressure was 77.4 ± 8.3 mmHg in men and 74.1 ± 9.1 mmHg in women. All P for trends across the age groups were significant (P < 0.001). All p-values for homogeneity among the age groups were also significant at p < 0.001 by analysis of variance (data not shown).

**Table 1:** Characteristics of subjects

		**Mean**	**(SD)**	**Mean**	**(SD)**	**Mean**	**(SD)**	**Mean**	**(SD)**	**Mean**	**(SD)**	**P for trend**
	Age (years)	-39		40-49		50-59		60-69		70-		
Men	n	1360		2768		3328		1891		527		
	VFA (cm^2^)	100.7***	(49.0)	118.7***	(51.8 )	125.6***	(53.9)	127.2***	(56.9)	128.6***	(57.4)	<0.001
	SFA (cm^2^)	146.4	(71.2)	144.3***	(63.7)	129.0***	(50.8)	117.8***	(44.4)	120.6***	(45.0)	<0.001
	Waist circumference (cm)	85.9***	(10.0)	87.0***	(9.2 )	86.4***	(8.4)	85.0	(8.2)	85.3	(8.8)	<0.001
	BMI (kg/m^2^)	24.3***	(3.5)	24.4***	(3.3)	23.9***	(2.8)	23.6*	(2.6 )	23.5*	(2.6)	<0.001
	VFA + SFA (cm^2^)	247.1***	(110.0)	263.0***	(104.6)	254.7*	(94.5)	245.0***	(91.9)	249.2**	(92.7)	0.007
	VFA/SFA (cm^2^)	0.7***	(0.3)	0.9***	(0.3)	1.0***	(0.4)	1.1***	(0.4)	1.1***	(0.4)	<0.001
	HbA1c (%)	5.6	(0.5)	5.7	(0.7)	5.9**	(0.8)	6.0***	(0.8)	6.0	(0.8)	<0.001
	Fasting plasma glucose (mg/dL)	100***	(13)	104***	(19)	109***	(23)	110***	(22)	109	(22)	<0.001
	Systolic blood pressure (mm Hg)	118***	(11)	120***	(12)	122***	(12)	125*	(13)	127	(12)	<0.001
	Diastolic blood pressure (mm Hg)	74***	(8)	77***	(9)	79***	(8)	79***	(8)	77***	(8)	<0.001
Women	n	133		291		595		587		90		
	VFA (cm^2^)	45.6	(33.1)	61.6	(41.6)	79.5	(41.6)	93.6	(46.7)	97.5	(48.4)	<0.001
	SFA (cm^2^)	152.4	(75.3)	169.6	(79.7)	185.2	(75.6)	187.0	(71.0)	186.5	(70.9)	<0.001
	Waist circumference (cm)	78.0	(9.0)	81.1	(10.0)	83.4	(9.3)	84.5	(9.6)	84.5	(8.7)	<0.001
	BMI (kg/m^2^)	21.6	(3.5)	22.7	(3.6)	23.0	(3.4)	23.2	(3.2)	22.8	(3.1)	<0.001
	VFA + SFA (cm^2^)	197.9	(103.5)	231.2	(113.8)	264.7	(108.0)	280.6	(106.6)	284.0	(107.0)	<0.001
	VFA/SFA (cm^2^)	0.3	(0.1)	0.4	(0.2)	0.4	(0.2)	0.5	(0.2)	0.5	(0.2)	<0.001
	HbA1c (%)	5.5	(0.3)	5.7	(0.9)	5.8	(0.9)	5.9	(0.6)	6.0	(0.6)	<0.001
	Fasting plasma glucose (mg/dL)	93	(7)	98	(22)	101	(24)	103	(17)	105	(18)	<0.001
	Systolic blood pressure (mm Hg)	111	(12)	114	(13)	120	(14)	124	(14)	127	(11)	<0.001
	Diastolic blood pressure (mm Hg)	69	(9)	71	(8)	75	(9)	76	(9)	75	(7)	<0.001

VFA; Visceral fat area, SFA; Subcutaneous fat area, BMI; Body mass index.

*P < 0.05, **P < 0.01, ***P < 0.001 for differences in mean values between men and women in each age group by *t*-test.

P for trend across the age groups was calculated by a linear regression analysis where the age group was coded as consecutive integers. All p-values for homogeneity among the age groups were also significant at p < 0.001 by analysis of variance (not shown in table).

Correlations between the VFA and the anthropometric indices are shown in Table 2. The VFA strongly correlated with WC and BMI in both men and women (r > 0.650), but the correlation coefficients were different in each age group.

**Table 2:** Correlation coefficients between the VFA and the anthropometric indices

	**Age**	**-39**	**40-49**	**50-59**	**60-69**	**70-**	**All**
Men	n	1360	2768	3328	1891	527	9874
	SFA	0.664	0.637	0.627	0.638	0.634	0.581
	WC	0.780	0.809	0.786	0.794	0.743	0.771
	BMI	0.694	0.681	0.678	0.686	0.685	0.650
Women	n	133	291	595	587	90	1696
	SFA	0.793	0.735	0.671	0.624	0.592	0.662
	WC	0.806	0.798	0.785	0.765	0.800	0.784
	BMI	0.808	0.773	0.761	0.710	0.714	0.727

Values are Pearson’s correlation coefficients.

All correlation coefficients were statistically significant at p < 0.001.

Correlations between the VFA plus SFA and the anthropometric indices are shown in Table 3. The VFA plus SFA strongly correlated with WC in both men and women (r > 0.901), but the correlation coefficients were different in each age group.

**Table 3:** Correlation coefficients between the VFA + SFA and the anthropometric indices

	**Age**	**-39**	**40-49**	**50-59**	**60-69**	**70-**	**All**
Men	n	1360	2768	3328	1891	527	9874
	WC	0.912	0.940	0.885	0.873	0.810	0.901
	BMI	0.869	0.860	0.825	0.803	0.808	0.838
Women	n	133	291	595	587	90	1696
	WC	0.953	0.933	0.954	0.935	0.959	0.945
	BMI	0.869	0.862	0.873	0.848	0.840	0.857

Values are Pearson’s correlation coefficients.

All correlation coefficients were statistically significant at p < 0.001.

**Figure 1:**
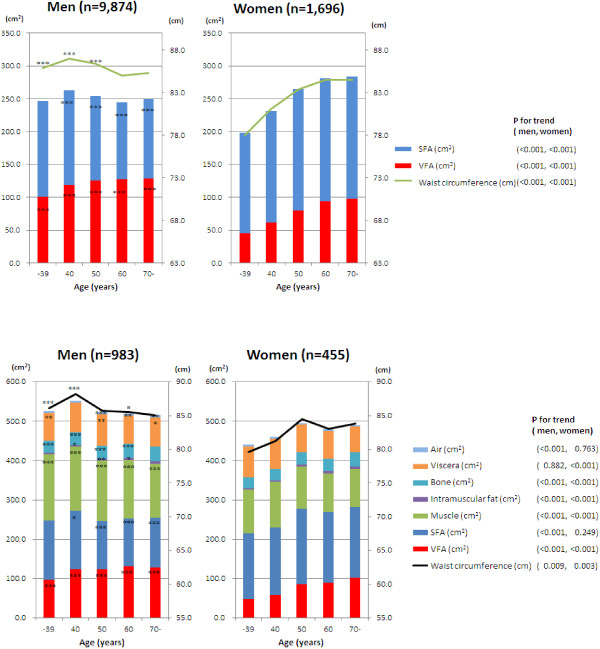
**Mean body components according to the age group measured by CT.** *P < 0.05, **P < 0.01, ***P < 0.001 for differences in mean values between men and women in each age group by *t*-test. P for trend across the age groups was calculated by a linear regression analysis where the age group was coded as consecutive integers.

Mean values of the VFA, SFA and WC within each age group are shown in Figure 1. In men, the mean WC decreased while the mean VFA paradoxically increased with age, thus showing almost no correlation between the mean WC and the mean VFA among the 5 age groups (r = -0.275, n = 5). However, a moderate correlation was shown between individuals’ WC and VFA (r = 0.771). When the VFA and SFA were summed, the pattern of the change in the mean WC and the mean VFA + SFA among the age groups became very similar (r = 0.915, n = 5) and the correlation between individuals’ WC and VFA + SFA became very strong (r = 0.901). Thus, it was demonstrated that the difference in the age-specific mean WC did not reflect the VFA alone, but represented the VFA + SFA because of the difference in the SFA among the age groups. In women, the association of age with the mean WC was very similar to the association of age with the mean VFA and mean VFA + SFA: the association being stronger with the latter (r = 0.979 and 0.996, n = 5). At the individual level, VFA + SFA was more strongly correlated to WC (r = 0.945) than VFA (r = 0.784). With respect to the differences between men and women, the ratios of WC to the VFA + SFA were greater in men than in women (i.e., the line of the mean WC was located at a higher position in men than in women). To clarify the reason for the greater ratios in men, we measured body components (VFA, SFA, muscle, intramuscular fat, bone, viscera, and air) in randomly selected men (n = 983) and women (n = 455). As shown in Figure 1, the muscle area was larger in men than in women in any age group; bone area was slightly larger in men than in women; and viscera were similar between men and women. It was clearly shown that the difference in WC to the VFA + SFA was due to the larger muscle area in men.

**Figure 2:**
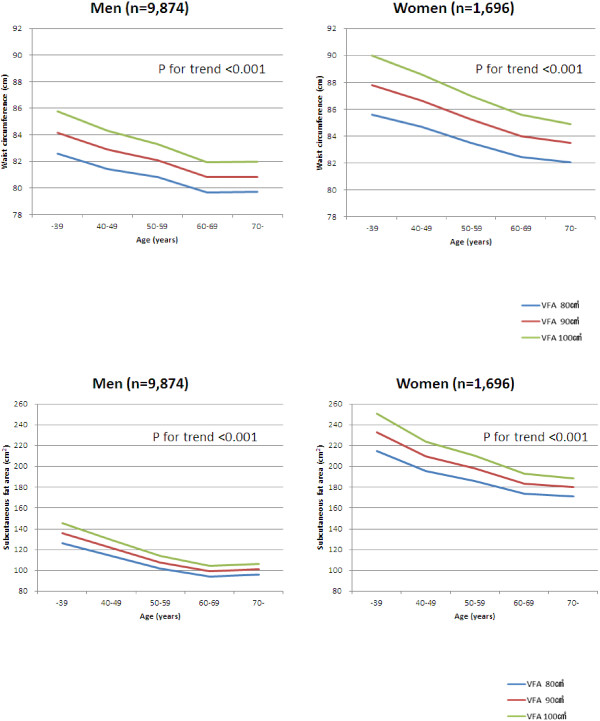
**WC or SFA values, corresponding to VFA values, stratified by age group.** P for trend across the age groups was calculated as the statistical significance of the partial regression coefficient of an age group (coded as consecutive integers) in a multiple linear regression analysis, where the dependent variable was WC (or SFA) and the independent variables were VFA and age group.

The difference in the WC values in each age group, with corresponding VFA values of 80 cm^2^, 90 cm^2^, and 100 cm^2^, is shown in Figure 2. In both men and women, the older generations had lower WC values compared to the younger generations when the VFA values were the same.

## Discussion

The present study showed that, as the generations become older, the VFA tended to increase in both men and women. However, the differences in the WC values in the age groups did not coincide with the VFA values, but did correlate with the difference in the sum of the VFA plus SFA in both genders. In the current criteria for metabolic syndrome, we used a common cut-off value for the WC for all ages. When using this cut-off threshold, it raises a problem in that the VFA for the younger generations is underestimated in both genders. Therefore, different cut-off values for the WC according to age should be considered.

There is no study which have evaluated the sex- and age-specific differences in body composition in large populations using more ‘direct’ methods, such as CT. Our study is the first report describing the body composition by age and sex using CT. In a previous study, Ito et al. determined body composition by DXA for the whole body and three anatomical regions: arms, legs, and trunk [15]. They reported curvilinear relationships in men for variables associated with adiposity, i.e. the BMI, WC, total or regional FM and % FM, with peaks observed in the forties age group. In women, these variables increased linearly in older subjects. The trends of the BMI and WC with age were similar to the results of our study. Although an age-related decline in lean mass was described [18], studies on large populations are scarce [19]. Furthermore, we expanded the calculations of the VFA and SFA separately using CT. The WC has been used as a simple index to measure the VFA in criteria used to diagnose metabolic syndrome [6]. However, this study showed that WC values corresponding to certain VFA values differed in each generation. Thus, a uniform value for the WC should not be used.

Furthermore, Ito et al. calculated the percentage fat mass of the whole body (% FM) as 100 × (FM)/(FM + lean mass + bone mineral content) [15]. They showed that the % FM in every generation was about 1.5-fold higher in women than in men. The results of the present study showed that in the older generations, men and women in their 60s or over, had similar WC values, but the sum of the VFA plus SFA differed between the genders. The sum of the fat content (VFA + SFA) was greater in women than in men, while the physical size of the viscera did not differ largely between the genders. We clarified that the muscle area contributed to the men’s greater WC. Our results were consistent with previously reported results. The correspondence of WC to VFA also differed by sex and age group. For example, for the women in the youngest group, the WC corresponding to a VFA of 100 cm^2^ was about 90 cm, whereas this was about 85 cm in the most elderly group: a discrepancy of about 5 cm. Similarly, in men, the elderly group had mean WC values about 4 cm smaller than the younger group.

The present study has several strengths. One of its strengths is the direct assessment of the VFA, SFA, WC, muscle, intramuscular fat, viscera, and bone using CT scanning. This allowed for precise determination of WC component. This is the first study to analyze the WC component using CT. In addition, the sample size of our study was extremely large (> 11,000 subjects). Nevertheless, the current study also has a limitation: the study subjects were chosen from a particular place (Hitachi Company) which is not representative of the general population. However, we confirmed similar mean BMI of each age group between our data and the National Nutrition Survey in Japan [20] (data not shown), suggesting our results could be used to be indicative of the general population.

## Conclusions

We concluded that differences in the values of WC by generational age did not coincide with the VFA. However, differences in the values of the sum of the VFA plus SFA did correlate with WC in both genders. The WC has been used as a simple index to measure the VFA in the criteria used to diagnose metabolic syndrome, but a WC value corresponding to a certain VFA value differed significantly by generational age. Thus, revised optimal cutoff values for the WC may be needed for each generation.

## Competing interest

The authors declare that they have no competing interest.

## Authors’ contributions

The principal investigator is YM, National Center for Global Health and Medicine. YM takes full responsibility for the work as a whole, including the study design, access to data, and the decision to submit and publish the manuscript. YM, TN, MS, and SY researched the data. YM, TN, YT, TM, TY, and MN contributed to discussions. YM wrote the manuscript. YM, TN, SY, TM, TY, and MN reviewed and edited the manuscript. All authors read and approved the final manuscript.

## References

[B1] Matsuzawa Y Metabolic syndrome-definition and diagnostic criteria in Japan J Jpn Soc Int Med 2005 94 188 203 10.5551/jat.12.301 16394611

[B2] Lakka HM Laaksonen DE Lakka TA Niskanen LK Kumpusalo E Tuomilehto J Salonen JT The metabolic syndrome and total and cardiovascular disease mortality in middle-aged men JAMA 2002 288 2709 2716 10.1001/jama.288.21.2709 12460094

[B3] Isomaa B Almgren P Tuomi T Forsén B Lahti K Nissén M Taskinen MR Groop L Cardiovascular morbidity and mortality associated with the metabolic syndrome Diabetes Care 2001 24 683 689 10.2337/diacare.24.4.683 11315831

[B4] Grundy SM Brewer HB Jr Cleeman JI Smith SC Jr Lenfant C American Heart Association, National Heart, Lung, and Blood Institute Definition of metabolic syndrome: report of the National Heart, Lung, and Blood Institute/American Heart Association conference on scientific issues related to definition Circulation 2004 109 433 438 10.1161/01.CIR.0000111245.75752.C6 14744958

[B5] World Health Organization Mondiale De La Santé 2011 Department of Measurement and Health Information [article online], April 2011. [http://apps.who.int/gho/data/node.main.1005?lang=en]

[B6] Grundy SM Cleeman JI Daniels SR Donato KA Eckel RH Franklin BA Gordon DJ Krauss RM Savage PJ Smith SC Jr Spertus JA Costa F American Heart Association; National Heart, Lung, and Blood Institute Diagnosis and management of the metabolic syndrome: an American Heart Association/National Heart, Lung, and Blood Institute Scientific Statement Circulation 2005 112 2735 2752 10.1161/CIRCULATIONAHA.105.169404 16157765

[B7] Takami H Nakamoto M Uemura H Katsuura S Yamaguchi M Hiyoshi M Sawachika F Juta T Arisawa K Inverse correlation between coffee consumption and prevalence of metabolic syndrome: baseline survey of the Japan Multi-Institutional Collaborative Cohort (J-MICC) study in Tokushima, Japan J Epidemiol 2013 23 12 20 10.2188/jea.JE20120053 23047663 PMC3700235

[B8] Xi B He D Hu Y Zhou D Prevalence of metabolic syndrome and its influencing factors among the Chinese adults: the China Health and Nutrition Survey in 2009 Prev Med 2013 in press 10.1016/j.ypmed.2013.09.023 PMC4044099 24103567

[B9] World Health Organization Definition, Diagnosis and Classification of Diabetes and its Complications: Report of a WHO Consultation 1999 Geneva: World Health Org WHO/NCD/NCS/99.2

[B10] International Diabetes Federation IDF Worldwide Definition of the Metabolic Syndrome [Article Online] 2005 [http://www.idf.org/node/1271?unode=1120071E-AACE-41D2-9FA0-BAB6E25BA072] Accessed Feb 3, 2014

[B11] Shiwaku K Nogi A Kitajima K Anuurad E Enkhmaa B Yamasaki M Kim JM Kim IS Lee SK Oyunsuren T Yamane Y Prevalence of the metabolic syndrome using the modified ATP III definitions for workers in Japan, Korea and Mongolia J Occup Health 2005 47 126 135 10.1539/joh.47.126 15824477

[B12] Examination Committee of Criteria for ‘Obesity Disease’ in Japan; Japan Society for the Study of Obesity New criteria for ‘obesity disease’ in Japan Circ J 2002 66 987 992 10.1253/circj.66.987 12419927

[B13] Oka R Miura K Sakurai M Nakamura K Yagi K Miyamoto S Moriuchi T Mabuchi H Yamagishi M Takeda Y Hifumi S Inazu A Nohara A Kawashiri MA Kobayashi J Comparison of waist circumference with body mass index for predicting abdominal adipose tissue Diabetes Res Clin Pract 2009 83 100 105 10.1016/j.diabres.2008.10.001 19019478

[B14] Yoshiike N Seino F Tajima S Arai Y Kawano M Furuhata T Inoue S Twenty-year changes in the prevalence of overweight in Japanese adults: the National Nutrition Survey 1976–95 Obes Rev 2002 3 183 190 10.1046/j.1467-789X.2002.00070.x 12164470

[B15] Ito H Ohshima A Ohto N Ogasawara M Tsuzuki M Takao K Hijii C Tanaka H Nishioka K Relation between body composition and age in healthy Japanese subjects Eur J Clin Nutr 2001 55 462 470 10.1038/sj.ejcn.1601206 11423923

[B16] Fox CS Massaro JM Hoffmann U Pou KM Maurovich-Horvat P Liu CY Vasan RS Murabito JM Meigs JB Cupples LA D’Agostino RB Sr O’Donnell CJ Abdominal visceral and subcutaneous adipose tissue compartments: association with metabolic risk factors in the Framingham Heart Study Circulation 2007 116 39 48 10.1161/CIRCULATIONAHA.106.675355 17576866

[B17] Nakagawa T Yamamoto S Irokawa M Development of the automated diagnosis CT screening system for visceral obesity Asian Pacific Journal of Disease Management 2008 2 31 38 10.7223/apjdm.2.31

[B18] Forbes GB Human Body Composition 1987 New York: Springer

[B19] Rico H Revilla M Villa LF Ruiz-Contreras D Hernández ER Alvarez de Buergo M The four-compartment models in body composition: data from a study with dual-energy X-ray absorptiometry and near-infrared interactance on 815 normal subjects Metabolism 1994 43 417 422 10.1016/0026-0495(94)90069-8 8159096

[B20] Division of Health Promotion and Nutrition, Ministry of Health, Labour and Welfare Annual report of the National Nutrition Survey in 2006 2008 Tokyo: Daiichi Publishing Co in Japanese

